# Beyond Crizotinib: A Systematic Review and Meta-Analysis of the Next-Generation ALK Inhibitors as First-Line Treatment for ALK-Translocated Lung Cancer

**DOI:** 10.3389/fonc.2022.921854

**Published:** 2022-06-14

**Authors:** Emilio Francesco Giunta, Alessio Signori, Howard Jack West, Giulio Metro, Alex Friedlaender, Kaushal Parikh, Giuseppe Luigi Banna, Alfredo Addeo

**Affiliations:** ^1^ Candiolo Cancer Institute, FPO-IRCCS, Candiolo (Turin), Italy; ^2^ Department of Health Sciences, University of Genova, Genoa, Italy; ^3^ Department of Medical Oncology and Therapeutics Research, City of Hope Comprehensive Cancer Center, Duarte, CA, United States; ^4^ Medical Oncology, Santa Maria Della Misericordia Hospital, Azienda Ospedaliera di Perugia, Perugia, Italy; ^5^ Oncology Department, University Hospital Geneva, Geneva, Switzerland; ^6^ Hackensack University Medical Center, Hackensack, NJ, United States

**Keywords:** lung cancer, ALK translocation, ALK inhibitors, crizotinib, first-line therapy

## Abstract

**Background:**

Second and third-generation ALK inhibitors (ALKIs) have been recently approved for ALK-translocated lung cancer treatment, improving - and expanding - the first-line scenario.

**Methods:**

In this systematic review and metanalysis, we investigated the efficacy and safety of next-generation ALKIs in untreated advanced ALK-translocated lung cancer patients, searching for randomized phase III controlled trials through databases (PubMed, EMBASE, and the Cochrane Library). Inclusion and exclusion of studies, quality assessment, data extraction, and synthesis were independently accomplished by two reviewers, with discrepancies adjudicated by a third reviewer. Stata (StataCorp., v.16) software was used for the metanalysis.

**Results:**

In total, seven randomized controlled trials met our inclusion criteria. Comparing the results of next-generation ALKIs and control therapy (crizotinib or chemotherapy), next-generation ALKIs significantly improved progression-free survival (PFS), overall survival (OS), objective response rate (ORR), disease control rate (DCR), any lesion (aCNSRR) and measurable lesions of central nervous system response rate (mCNSRR). Safety results were similar between the experimental and control groups.

**Conclusion:**

Our analysis confirmed that next-generation ALKIs are the preferred first-line treatment option for ALK-translocated lung cancer. They are superior to crizotinib or chemotherapy in several clinical endpoints, including OS, PFS, ORR and CNS disease control, without increased toxicity. In the absence of head-to-head data, the choice between these molecules should be guided by physician experience and preference, drug-specific safety profile and schedule.

## 1 Introduction

Anaplastic lymphoma kinase (ALK) gene rearrangements have been found in up to 5% of non-small cell lung cancer (NSCLC) patients, mostly with adenocarcinoma histology, more frequently with young age and never- or light smoking history (1). ALK gene rearrangements result from the fusion between a partner gene (i.e., EML4, KIF5B, KLC1, etc.) and the ALK gene itself, thus determining the constitutive activation of the ALK kinase domain; they represent a driver mutation in several types of cancer, including NSCLC (2, 3).

The development of ALK inhibitors (ALKIs) has dramatically changed the management of patients with NSCLC harboring this alteration. Crizotinib was the first-in-class molecule approved for clinical use in ALK-translocated lung adenocarcinoma (4). Crizotinib prolonged progression-free survival (PFS) and improved objective response rate (ORR), but not overall survival (OS), when compared to standard platinum-based chemotherapy in both pretreated and treatment-naive patients with advanced ALK-positive lung cancer (5, 6). Second (i.e., ceritinib, alectinib, brigatinib) and third-generation (i.e., lorlatinib and ensartinib) ALKIs have therefore been developed to overcome crizotinib resistance and improve clinical outcomes, including central nervous system (CNS) penetration. Up to 30% of patients with tumors harboring ALK alterations present at diagnosis with brain metastases or will develop those while on treatment (7). Third-generation ALKIs were also developed to target specific ALK mutations like the G1202R, a common acquired resistance mutation to other ALKIs, including second-generation molecules (7).

Among next-generation ALKIs, ceritinib has not been compared head-to-head to crizotinib, but standard chemotherapy, and only an adjusted indirect comparison with crizotinib is currently available (8, 9).

Previous systematic reviews and meta-analyses of ALKIs for the treatment of NSCLC have highlighted improved OS and PFS of crizotinib and other ALKIs versus chemotherapy (10–12). None of the recently published meta-analyses on the same topic (13–15) has specifically investigated the role of next-generation ALKIs towards crizotinib or chemotherapy. As head-to-head comparisons between next-generation ALKIs are unavailable, we conducted a systematic review of randomized phase III controlled trials where untreated patients received a second or third-generation ALKI as the experimental treatment for advanced ALK-positive NSCLC. We investigated efficacy and safety outcomes through formal meta-analysis and addressed common questions for clinical decision-making.

## 2 Methods

We conducted a systematic literature review (SLR) following the Cochrane Handbook for Systematic Reviews for Interventions and the Preferred Reporting Items for Systematic Reviews and Meta-Analyses (PRISMA) checklist (16, 17). As this was not an individual patient-level meta-analysis, institutional review board permission and informed consent were not sought.

### 2.1 Study Objective

To describe the efficacy and safety of second and third-generation ALKIs as first-line treatment for patients with ALK translocated/rearranged NSCLC.

### 2.2 Eligibility Criteria

Randomized phase III controlled trials including adults (≥ 18 years) with stage IV squamous or non-squamous NSCLC with ALK translocation/rearrangement and no prior chemotherapy or targeted therapy for stage IV disease were included. Interventions of interest included monotherapy with next-generation ALKIs, namely ceritinib, alectinib, brigatinib, lorlatinib, ensartinib.

### 2.3 Data Source

The systematic literature search was carried out in December 2021 on Embase, PubMed, Cochrane Central Register of Controlled Trials (CENTRAL) and Cochrane Database of Systematic Reviews (CDSR) databases. Additional sources included reference lists from relevant publications and congress abstracts (2010-2021) from the American Society of Clinical Oncology (ASCO), the European Lung Cancer Congress (ELCC), the European Society for Medical Oncology (ESMO), and the World Conference on Lung Cancer (WCLC) of the International Association for the Study of Lung Cancer (IASLC). Full search terms and the details of the databases interrogated are provided in two tables, see **Supplemental Data 1**, **2**.

Outcomes of interest included: PFS (including subgroups analysis for age, sex, race, smoking status,

Performance Status [PS], and CNS involvement at baseline), overall survival (OS), ORR, disease control rate (DCR), CNS response rate (CNSRR, either any lesions - aCNSRR - or only measurable lesions - mCNSRR), and adverse events (AEs) (including grade [G] ≥3 AEs, serious AEs [SAEs], interruption due to AEs, and discontinuation due to AEs).

### 2.4 Study Selection and Data Extraction

Two independent reviewers (EFG and GLB) screened titles and abstracts of retrieved records and then the full texts of potentially eligible papers, with discrepancies adjudicated by a third reviewer (AA). Detailed data extraction and risk of bias (ROB) assessment were carried out by two independent reviewers (EFG and GLB), with discrepancies adjudicated by a third reviewer (AA). ROB was assessed using the Cochrane Collaboration’s ROB tool for RCTs (18).

### 2.5 Statistical Analysis

Pooled hazard ratios (HRs) for time-to-event outcomes (OS and PFS), and risk ratios (RRs) for dichotomous outcomes (ORR, DCR, CNSSRR, and safety outcomes), in both the experimental and controls arms, according to treatment type and in patients’ subgroups (only for PFS), were calculated. Regarding dichotomous outcomes and the RR calculation, studies not reporting the number of events were excluded from the analysis. A classic meta-analysis was conducted to estimate the experimental vs control arm pooled effect size for the time to event (pooled HR) and dichotomous outcomes (pooled RR). A minimum of two studies was required to proceed with data synthesis.

The study effect sizes were synthesized using random-effects models accounting for heterogeneity. Heterogeneity was quantified by the Higgins I^2^ coefficient and statistically tested by the Cochrane Q test.

P-values less than 0.05 were considered statistically significant. Stata (StataCorp., v.16) statistical software was used to compute.

## 3 Results

### 3.1 Study Selection and Characteristics

**Figure 1:**
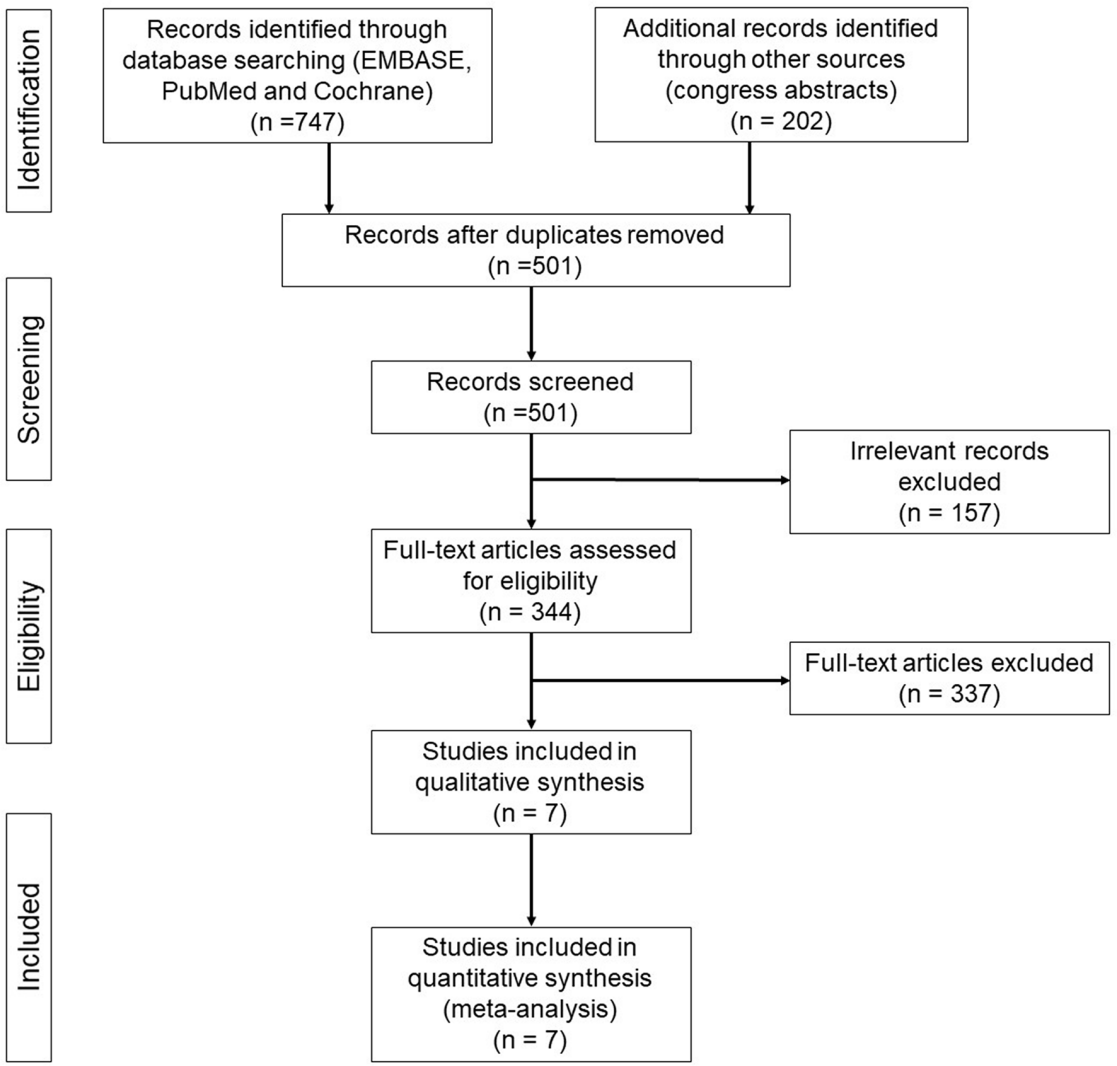
PRISMA flow diagram of randomized phase III clinical trials of next-generation ALKIs as first line treatment for advanced ALK-positive NSCLC.

Our initial literature search found a total of 747 relevant citations from electronic databases and 202 congress abstracts. After the exclusion of duplicates, 501 studies were included. Subsequently, 494 of 501 studies were excluded because their abstracts did not fulfil our inclusion criteria (**Figure 1**).

Seven trials met the eligibility criteria: ALEX, J-ALEX, ALTA-1L, ASCEND-4, ALESIA, eXalt3, and CROWN trial (8, 19–24). All those trials compared a next-generation ALKI to crizotinib except for the ASCEND-4 trial, which compared ceritinib to chemotherapy. The primary endpoint was PFS for all the seven studies, albeit with some differences in assessment (assessed by investigators in 2 trials and by independent review in 5 trials) and outcome reporting (median PFS in five trials and 1-year PFS rate in two trials). The main characteristics and outcomes of the seven trials are summarized in **Table 1**, while the other efficacy outcomes are reported in a different table (see **Supplemental Data 3**); safety data are reported in **Table 2**.

**Table 1:** Characteristics and main outcomes of selected randomized clinical trials in patients with ALK-translocated lung cancer.

Name of the trial	First author and year of publication	Treatment in the control arm: drug/dose (n of pts)	Treatment in the experimental arm: drug/dose (n of pts)	Median follow-up time in the control arm	Median follow-up time in the experimental arm	Primary endpoint	PFS control vs experimental arm		PFS: HR (95% CI) and p value (experimental vs control)	OS: HR (95% CI) and p value (experimental vs control)
ALEX (19)	Peters et al., 2017	crizotinib 250 mg BID (151)	alectinib 600 mg BID (152)	17.6 months	18.6 months	PFS (IA)	1y PFS rate; 48.7% (95% CI: 40.4-56.9%)	1y PFS rate: 68.4% (95% CI: 61-75.9%)	0.47 (95% CI: 0.34-0.65). p<0.001	0.76 (95% CI: 0.48-1.2). p=0.24
J-ALEX (20)	Hida et al., 2017	crizotinib 250 mg BID (104)	alectinib 300 mg BID (103)	12.2 months	12 months	PFS (IRF)	Median: 10.2 months (range: 8.2-12.0)	Median: NE (range: 20.3 months-NE)	0.34 (95% CI: 0.17-0.71). p<0.0001	NA
ALTA-1L (21)	Camidge et al., 2020	crizotinib 250 mg BID (137)	brigatinib 180 mg QD (with 7-day lead-in at 90 mg QD) (136)	NA	24.9 months	PFS (BIRC)	Median: 11.0 months (range: 9.2-12.9)	Median: 24.0 months (range: 18.5-NR)	0.49 (95% CI: 0.35-0.68). p=0.0001	0.92 (95% CI: 0.57-1.47). p=0.771
ASCEND-4 (8)	Soria et al., 2017	platinum-based CT: cisplatin 75 mg/m2 or carboplatin AUC 5-6 + pemetrexed 500 mg/m2 q3w for 4 cycles followed by pemetrexed maintenance (175)	ceritinib 750 mg QD (189)	NA	NA	PFS (BIRC)	Median: 8.1 months (range: 5.8-11.1)	Median: 16.6 months (range: 12.6-27.2)	0.55 (95% CI: 0.42-0.73). p<0.00001	0.73 (95% CI: 0.5-1.08). p=0.056
ALESIA (22)	Zhou et al., 2019	crizotinib 250 mg BID (62)	alectinib 600 mg BID (125)	15 months	16.2 months	PFS (IA)	Median: 11.1 months (range: 9.1-13.0)	Median: NE (range: 9,1 months-NE)	0.22 (95% CI: 0.13-0.38). p<0.0001	0.28 (95% CI: 0.12-0.68). p=0.0027
eXalt3 (23)	Horn et al., 2021	crizotinib 250 mg BID (146)	ensartinib 225 mg QD (143)	20.2 months	23.8 months	PFS (BIRC)	Median: 12.7 months (range: 0.03-38.6)	Median: 25.8 months (range: 0.03-44.0)	0.51 (95% CI: 0.35-0.72). p<0.001	0.91 (95% CI: 0.54-1.54). p=0.73
CROWN (24)	Shaw et al., 2020	crizotinib 250 mg BID (142)	lorlatinib 100 mg QD (149)	14.8 months	18.3 months	PFS (BIRC)	1y PFS rate; 39% (95% CI: 30-48%)	1y PFS rate: 78% (95% CI: 70-84%)	0.28 (95% CI: 0.19-0.41). p<0.001	0.72 (95% CI: 0.41-1.25). p= NA

BID, bis in die; BIRC, blinded independent review committee; CI, confidence interval; IA, investigator assessed; IRF, assessed by independent review facility; NA, not available; NE, not estimable; NR, not reached; OS, overall survival; PFS, progression free survival; QD, quaque die.

**Table 2:** Safety data from the selected randomized clinical trials in patients with ALK-translocated lung cancer.

Name of the trial	First author and year of publication	Treatment in the control arm: drug/dose	Treatment in the experimental arm: drug/dose	No of pts in the control arm	No of pts in the experimental arm	% of patients developing G≥3 AEs: control vs experimental arm		% of patients developing SAEs: control vs experimental arm		% of patients developing fatal AEs: control vs experimental arm		% of patients discontinuing drugs due to AEs: control vs experimental arm		% of patients needing dose reduction due to AEs: control vs experimental arm		% of patients interrupting drugs due to AEs: control vs experimental arm	
ALEX (19)	Peters et al., 2017	crizotinib 250 mg BID	alectinib 600 mg BID	151	152	50%	41%	29%	28%	5%	3%	13%	11%	21%	16%	25%	19%
J-ALEX (20)	Hida et al., 2017	crizotinib 250 mg BID	alectinib 300 mg BID	104	103	52%	26%	NA	NA	0	0	20%	9%	NA	NA	74%	29%
ALTA-1L (21)	Camidge et al., 2020	crizotinib 250 mg BID	brigatinib 180 mg QD (with 7-day lead-in at 90 mg QD)	137	136	61%	73%	NA	NA	NA	NA	9%	13%	25%	38%	NA	NA
ASCEND-4 (8)	Soria et al., 2017	platinum-based CT: cisplatin 75 mg/m2 or carboplatin AUC 5-6 + pemetrexed 500 mg/m2 q3w for 4 cycles followed by pemetrexed maintenance	ceritinib 750 mg QD	175	189	62%	78%	NA	NA	NA	NA	11%	5%	NA	NA	NA	NA
ALESIA (22)	Zhou et al., 2019	crizotinib 250 mg BID	alectinib 600 mg BID	62	125	43%	27%	26%	15%	5%	2%	10%	7%	23%	24%	27%	26%
eXalt3 (23)	Horn et al., 2021	crizotinib 250 mg BID	ensartinib 225 mg QD	146	143	40%	50%	6% (TR)	8% (TR)	3%	1%	7%	9%	20%	24%	NA	NA
CROWN (24)	Shaw et al., 2020	crizotinib 250 mg BID	lorlatinib 100 mg QD	142	149	56%	72%	27%	34%	5%	5%	9%	7%	15%	21%	47%	49%

AEs, adverse events; BID, bis in die; CI, confidence interval; G, grade; IA, investigator assessed; NA, not available; QD, quoque die; SAEs, serious advent events; TR, treatment-related.

### 3.2 Efficacy Outcomes by the Systematic Review and Meta-Analysis

#### 3.2.1 Progression-Free Survival

**Figure 2:**
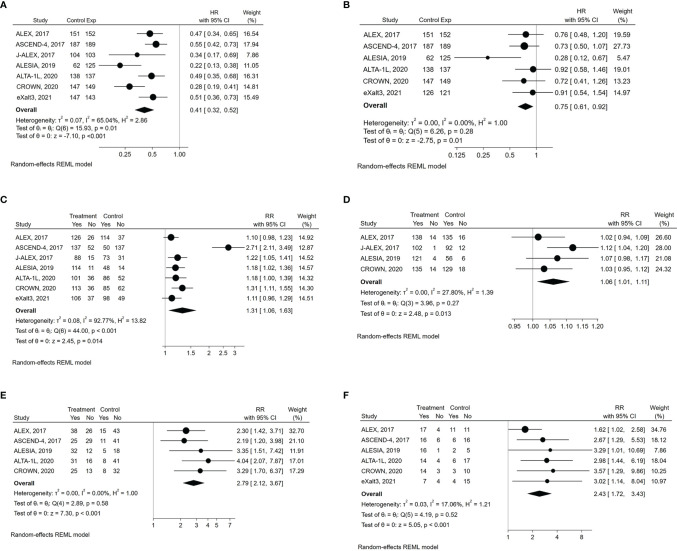
Forest plots of HR (hazard ratio) and RR (relative risk) for efficacy outcomes associated with next-generation ALKIs compared to control therapies. **(A)** PFS (progression‐free survival). **(B)** OS (overall survival). **(C)** ORR (overall response rate). **(D)** DCR (disease control rate). **(E)** aCNSRR (any lesion of central nervous system response rate). **(F)** mCNSRR (measurable lesion of central nervous system response rate).

The experimental drugs significantly prolonged PFS compared to either crizotinib or chemotherapy in all the studies (**Table 1**) (8, 19–24). Compared to the control therapy, treatment with next-generation ALKIs significantly improved PFS among all participants (HR: 0.41, 95% CI: 0.32-0.52, p >0.01; I^2^ = 65%, p for heterogeneity = 0.0142) (**Table 3**) (**Figure 2A**).

**Table 3:** Pooled results for the efficacy and safety outcomes.

Efficacy outcomes	HR (95% CI)	p-value	Heterogeneity
PFS	0.41 (0.32-0.52)	<0.001	I^2 =^ 65%; p= 0.0142
OS	0.75 (0.62-0.92)	0.006	I^2 =^ 0%; p= 0.28
**Other efficacy outcomes**	**RR (95% CI)**	**p-value**	**Heterogeneity**
ORR	1.31 (1.06-1.63)	0.014	I^2 =^ 92.8%; p<0.001
DCR	1.06 (1.01-1.11)	0.013	I^2 =^ 27.8%; p= 0.26
aCNSRR	2.79 (2.12-3.67)	<0.001	I^2 =^ 0%; p=0.58
mCNSRR	2.43 (1.72-3.43)	<0.001	I^2 =^ 17.1%; p= 0.52
**Safety outcomes**	**RR (95% CI)**	**p-value**	**Heterogeneity**
All grade AEs	1.003 (0.990-1.016)	0.70	I^2 =^ 27.4%; p= 0.25
Grade 3-4 AEs	0.96 (0.74-1.26)	0.79	I^2 =^ 90.5%; p<0.001
SAEs	0.99 (0.72-1.37)	0.96	I^2 =^ 44.9%; p= 0.14
Fatal AEs	0.65 (0.34-1.23)	0.19	I^2 =^ 0%; p= 0.67
Discontinuation rate due to AEs	0.79 (0.56-1.12)	0.18	I^2 =^ 34.4%; p= 0.18
Dose reduction rate due to AEs	1.18 (0.92-1.51)	0.19	I^2 =^ 32.1%; p= 0.22
Dose interruption rate due to AEs	0.74 (0.47-1.16)	0.18	I^2 =^ 84.7%; p<0.001

aCNSRR, any lesion of central nervous system response rate; AEs, adverse events; DCR, disease control rate; HR, hazard ratio; mCNSRR, measurable lesion of central nervous system response rate; ORR, overall response rate; OS, overall survival; PFS, progression free survival; RR, relative risk; SAEs, serious advent events; TR, treatment-related.

**Figure 3:**
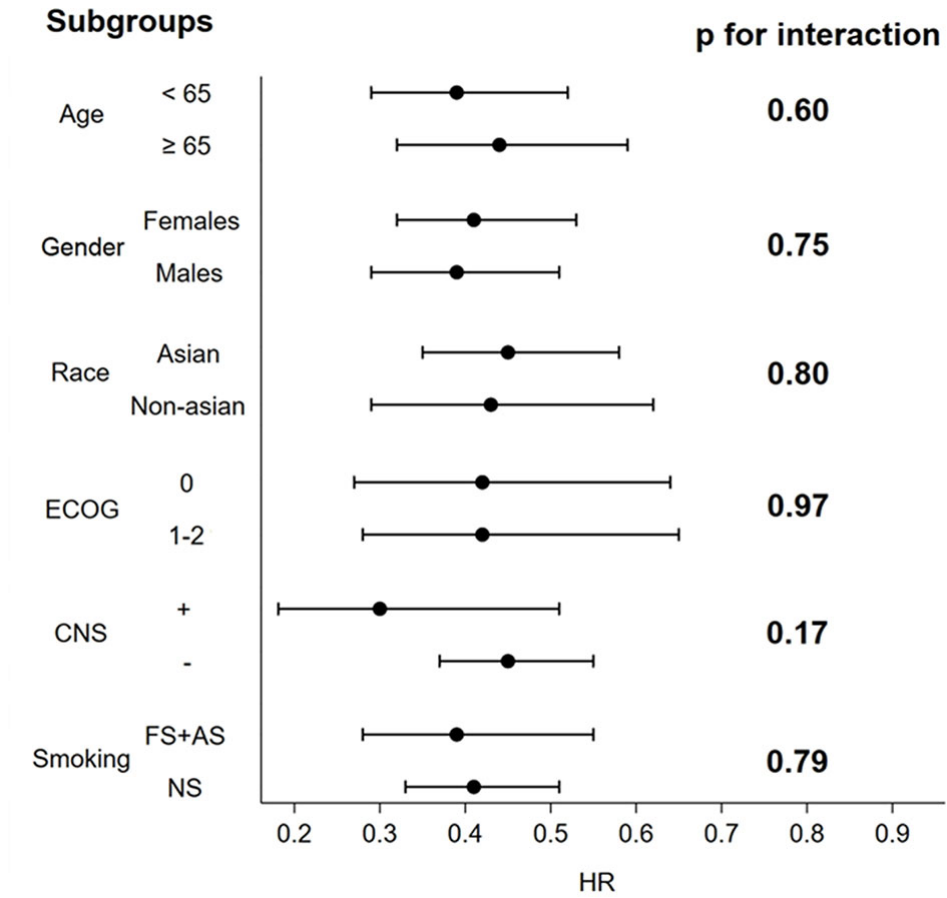
Forest plots of HR associated with next-generation ALKIs compared to control therapies for prespecified PFS subgroups analysis.

At the subgroup analyses of PFS by age, sex, race, smoking status, PS, baseline CNS involvement status, no significant interactions were observed in any subgroup, thus confirming the overall significant PFS benefit from second and third-generation ALKIs (see **Supplemental Data 4**, **5**) (**Figure 3**). Notably, there were some differences in the subgroup analysis among the included trials, i.e., the inclusion of Asian patients only in the J-ALEX and ALESIA trials and the age threshold of 75 years in the J-ALEX trial (see **Supplemental Data 4**).

#### 3.2.2 Overall Survival and Response Rates

OS analysis results were available for six of the seven trials at their first in-extenso publication (Table, see **Supplemental Data 3**) (8, 19, 21–24). Statistically significant improvement in OS was observed only in the ALESIA trial (HR: 0.28, 95% CI: 0.12-0.68, p= 0.0027), while in the others, the lack of OS difference was mainly due to immature data for analysis and the confounding effect of crossover (8, 19–24). Nevertheless, by the pairwise meta-analysis, we found a significant OS benefit from next-generation ALKIs over control therapies (HR: 0.75, 95% CI: 0.62-0.92, p = 0.006, I^2^ = 0%, p for heterogeneity = 0.28) (**Figure 2B**).

Regarding response rates, ORR results were available for all the trials, though only four reported DCR results (19, 20, 22, 24). Intracranial responses of measurable lesions (mCNSRR) were assessed in all trials except for J-ALEX, whilst the assessment of any intracranial lesions, whether measurable or not (aCNSRR), was not available in the J-ALEX and eXalt3 trials (20, 24). ORR (relative risk, RR: 1.31, 95% CI: 1.06-1.63, p= 0.014; I^2^ = 92.8%, p<0.001) and DCR (RR: 1.06, 95% CI: 1.01-1.11, p= 0.013; I^2^ = 27.8%, p= 0.26, respectively) were both higher with the next-generation ALKIs than controls, albeit with a different heterogeneity index (**Figures 2C, D**). There was consistency between aCNSRR and mCNSRR, with a greater benefit from next-generation ALKIs for both parameters (**Figures 2E, F**).

### 3.3 Safety Outcomes by the Systematic Review

**Figure 4:**
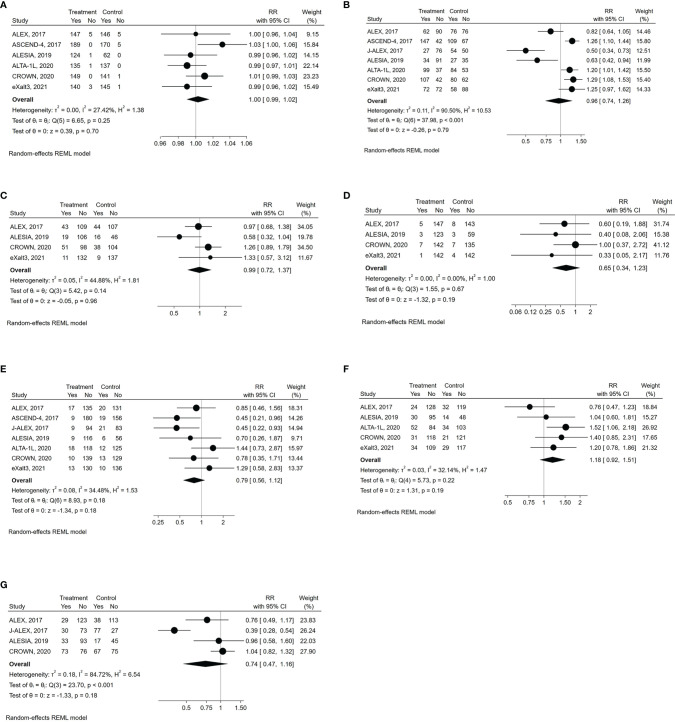
Forest plots of RR (relative risk) for safety outcomes associated with next-generation ALKIs compared to control therapies. **(A)** all grade AEs (adverse events). **(B)** G3-4 AEs. **(C)** SAEs (serious adverse events). **(D)** fatal AEs. **(E)** drug discontinuation due to AEs. **(F)** dose reduction due to AEs. **(G)** dose interruption due to AEs.

All grade AEs were available for all the examined trials except the J-ALEX, while G≥3 AEs were reported in all of them (**Table 2**) (8, 19–24). Compared with control therapy, the use of a next-generation ALKI was associated with neither an increased risk of all grade (RR: 1.003, 95% CI: 0.990-1.016, p= 0.70; I^2^ = 27.4%, p for heterogeneity = 0.25) nor G≥3 AEs (RR: 0.96, 95% CI: 0.74-1.26, p= 0.79; I^2^ = 90.5%, p for heterogeneity <0.001) (**Figures 4A, B**).

SAEs were reported only in four trials (**Table 2**), with similar results between next-generation ALKIs and control arms (RR: 0.99, 95% CI: 0.72-1.37, p= 0.96; I^2^ = 44.9%; p for heterogeneity = 0.14) (19, 22–24). Fatal AEs (reported in five trials) were also similar (RR: 0.65, 95% CI: 0.34-1.23, p= 0.19; I^2^ = 0%; p for heterogeneity = 0.67) (**Figures 4C, D**) (19, 20, 22–24).

All the trials reported the AE-related drug discontinuation, five the dose reduction and four the dose interruption rates. There were non-significant differences between experimental and control arms for all those parameters (**Table 2**) (**Figures 4E‐G**) (8, 19–24).

### 3.4 Risk of Bias

Overall, the trials were deemed at low risk for bias concerning blinding of outcome assessment (Figure, see **Supplemental Data 6**). A high risk of bias was found for blinding participants and research personnel, and a high percentage of unclear risk for random sequence generation, allocation concealment and selective reporting.

## 4 Discussion

Several ALKIs have been developed and tested to overtake crizotinib in the past few years. This first-in-class molecule is characterized by the onset of resistance in a high percentage of patients (25). Moreover, crizotinib leads to a sub-optimal intracranial response rate, explained by its low penetration through the blood-brain barrier (26, 27). Second and third-generation ALKIs initially demonstrated efficacy in crizotinib-pretreated patients, mainly because of their activity against resistant clones arising from crizotinib treatment and intracranial efficacy (28). Therefore, moving to the first-line setting was an obvious transition for those molecules, given the possibility of delaying the onset of resistance and providing deeper extra- and intracranial responses than crizotinib, which effects were likely to translate into longer survival.

The PFS advantage of next-generation ALKIs relative to crizotinib or chemotherapy was clear and maintained in all the examined subgroups, underlining the superiority of this drug class. ORR and DCR were also enhanced by next-generation ALKIs, justifying their use as first-line treatment for patients with ALK-translocated NSCLC. Concerning intracranial responses, both measurable and non-measurable lesions were significantly responsive to next-generation ALKIs, confirming their superior intracranial activity compared to crizotinib or chemotherapy.

Our meta-analysis highlighted a statistically significant improvement in OS with a very low heterogeneity among trials, albeit with several caveats. First, the OS data from all the trials are still immature, meaning that we cannot infer the real impact of the use of next-generation ALKIs, especially over the long term. Second, we must recognize that the variable crossover (allowed in the ALTA-1L and ASCEND-4 trials; after study withdrawal, in the J-ALEX and ALESIA trials; not allowed in the ALEX, eXalt3, and CROWN trials) has relevant consequences on the interpretability of results, since the therapeutic sequence may profoundly influence OS (8, 19–24, 29). Notably, even with the crossover option, a sizeable number of patients in the control arm may not proceed to receive any subsequent line of therapy on progression and clinical deterioration. In the ALEX, 36.8% of patients who had progressive disease on crizotinib did not receive any following line of treatment. These findings support the general concept that administering the most effective treatment upfront would be the optimal strategy, given the risk of attrition throughout progression, especially in a disease at high risk of intracranial progression.

Our meta-analysis did not identify any statistically significant difference between next-generation ALKIs and control therapies concerning safety data. However, it is noteworthy that each ALKI shows a different safety profile and specific AEs, with varying consequences on patients’ health. The higher dose reduction rates observed with next-generation ALKIs probably reflect the clinical need to find the appropriate drug dose for each patient, as it was not associated with an evident decrease in drug efficacy. We conclude that second and third-generation ALKIs do not demonstrate a worse toxicity profile than control therapies, but clinicians should consider that specific AEs in choosing among them.

Heterogeneity was found in the analysis of PFS, ORR, AEs, and dose interruption rate due to AEs, as reported in **Table 3**. As the expression of the intervention effects variability, heterogeneity was mainly related to the different efficacy and safety profile of ALKIs. Heterogeneity in PFS (I^2^ = 65%; p= 0.0142) could also be explained by the different trial follow-ups at the time of their analysis. The high heterogeneity observed in the ORR analysis (I^2^ = 92.8%; p<0.001) relies on the outlier value from the ASCEND-4 trial. This effect is likely related to the chemotherapy control arm and the corresponding lower disease responses than TKIs. G3-4 AEs analysis has also revealed a high heterogeneity (I^2^ = 90.5%, p<0.001), mainly driven by the three trials adopting alectinib as the experimental arm. Based on the forest plot, we could cautiously conclude for a more favorable safety profile of alectinib than all the other ALKIs. The heterogeneity in the dose interruption rate due to AEs (I^2^ = 84.7%; p<0.001), related to the low number of events registered in patients treated with alectinib, may be corroborated this hypothesis.

As alectinib, brigatinib, ceritinib, and lorlatinib have been approved as frontline therapy, and ensartinib is on the path to its approval, careful consideration of the individual patient characteristics could drive the choice of the appropriate first-line ALKI. Ceritinib, for instance, has been associated with gastrointestinal side effects, including diarrhea, vomiting, or elevated liver enzymes in the majority of patients. These effects can be managed by administering ceritinib with food (30). Alectinib needs to be taken twice a day, warranting daily ingestion of eight capsules. It has also been associated with anemia, myalgia, and creatine kinase elevation, which can be functionally limiting for younger patients who prefer to remain active (19). While all ALK inhibitors have been causally associated with drug-related interstitial lung disease (ILD), brigatinib has the highest rate of ILD and is associated with early-onset pulmonary events (EOPEs) (31). These EOPEs are dose-related and justify the dosing strategy of brigatinib with a step-up dose after seven days if well tolerated. On the other hand, lorlatinib may yield G≥3 hyperlipidemia in about 20% of patients and peripheral edema in 10%. Lorlatinib has also been linked with cognitive side effects and mood changes in 21% and 16% of patients (24). However, its preclinical profile showed broad activity against several resistance mutations, including the EML4-ALK G1202R and L1196M.

Given the lack of head-to-head comparison between second-generation ALKIs and the 3^rd^ generation lorlatinib, its activity on acquired resistance mutations and the distinct safety profile, we would reserve lorlatinib for a subsequent line. While this consideration might crawl with the recommendation to use the most effective treatment upfront, we have to consider that tolerable and effective therapies are also essential for later lines of treatment. However, this field is continuously evolving with newer TKIs characterized by expanded activity like the TPX-0131 and NVL-655 currently under investigation and data on optimal sequencing likely be available shortly.

Regarding other therapies for ALK-translocated lung cancer, data about immune checkpoint inhibitors (ICIs) are scarce and suggest inferior activity of single-agent ICIs (28a). When ALKIs fail, chemotherapy remains the standard of care, with the addition of ICI currently unclear as per the IMpower 150 trial and requiring further prospective data (32).

There are several limitations related to our meta-analysis. First, we acknowledge the non-unique control therapy we considered (crizotinib in six trials and chemotherapy in the ASCEND-4). As noted above, we included the ASCEND-4, as ceritinib is a second-generation ALKI and crizotinib did not demonstrate an OS benefit over chemotherapy in the first-line setting. Another limitation is the exclusive enrollment of the Asian population in two trials, J-ALEX and ALESIA. Other limitations are the relatively immature OS data, the underreported data about serious and fatal events and the lack of individual patient data.

Other meta-analyses have been published on the same topic (13–15). Different from the others, we compared next-generation ALKIs to crizotinib or chemotherapy, or the standard of care before their approval. We considered the most recent publications, i.e. updated results from ALTA-1L and eXalt-3, differently from Peng et al. (13). Furthermore, in the analysis by Ma et al. (14), crizotinib was not considered as the comparison but included, alongside the next-generation ALKIs, as an option for first-line therapy.

## 5 Conclusions

The recent approval of second and third-generation ALKIs has dramatically changed the treatment algorithm of ALK-translocated lung cancer. In the absence of head-to-head comparisons between next-generation ALKIs, any next-generation ALKI may be considered a valid option as first-line therapy (33, 34). Our meta-analysis showed consistency among efficacy and safety outcomes, highlighting a class-effect improvement over control therapies. The choice of the preferred frontline ALKI mainly relies on the physician’s judgment based on personal experience and ALKI differences in safety profiles.

## Data Availability Statement

The original contributions presented in the study are included in the article/**Supplementary Material**. Further inquiries can be directed to the corresponding author.

## Author Contributions

GB and AA: conceptualization and supervision; EG and AS: formal analysis, writing original draft, methodology; AS: software. HW, GM, AF, and KP: validation and review and editing. All authors contributed to the article and approved the submitted version.

## Funding

GB’s work was supported by “FPRC 5xmille Ministero Salute 2017 PTCRC-Intra 2020 ‘CTU-Lung’”.

## Conflict of Interest

HW reports personal fees from Genentech/Roche, AstraZeneca, Merck, Takeda, and Bristol Myers Squibb; GM reports consultancies from Boehringer Ingelheim and Bristol-Myers Squibb and travel grants from AstraZeneca; AF reports personal fees from Roche, Pfizer, Astellas and Bristol Myers Squibb; GB reports personal fees from Janssen Cilag, Boehringer Ingelheim and Roche; AA reports personal fees from BMS, Astrazeneca, Roche, Pfizer, MSD, Boehringer;

The remaining authors declare that the research was conducted in the absence of any commercial or financial relationships that could be construed as a potential conflict of interest.

## Publisher’s Note

All claims expressed in this article are solely those of the authors and do not necessarily represent those of their affiliated organizations, or those of the publisher, the editors and the reviewers. Any product that may be evaluated in this article, or claim that may be made by its manufacturer, is not guaranteed or endorsed by the publisher.
