# Supplementary material for: Beyond Crizotinib: A Systematic Review and Meta-Analysis of the Next-Generation ALK Inhibitors as First-Line Treatment for ALK-Translocated Lung Cancer
Source: Front Oncol. 2022 Jun 14;12:921854. doi: 10.3389/fonc.2022.921854 (PMC9239548; doi:10.3389/fonc.2022.921854)
Supplement: Supplementary file 1 [file DataSheet_1.docx]

**SUPPLEMENTAL DATA**

| Step | Search | EMBASE (n. of results) | PubMed (n. of results) | Cochrane Library (n. of results) |
| --- | --- | --- | --- | --- |
| 1 | (non small cell* OR nonsmall cell* OR large cell* OR squamous cell* OR squamous small cell* OR nonsquamous cell* OR nonsquamous small cell* OR epidermoid) AND (cancer* OR carcin* OR tumor* OR tumour* OR neoplas* OR oncol* OR malignan*) |  |  |  |
| 2 | (lung OR lungs OR pulmonary OR bronchial OR bronchus) |  |  |  |
| 3 | 1 AND 2 |  |  |  |
| 4 | (lung OR lungs OR pulmonary OR bronchial) AND (adenocarcin* OR adenocancer*) |  |  |  |
| 5 | NSCLC* |  |  |  |
| 6 | 3 OR 4 OR 5 | 282433 | 158904 | 1849491 |
| 7 | (randomised OR randomized OR controlled) AND (clinical trial OR trial) |  |  |  |
| 8 | (anaplastic lymphoma kinase OR ALK OR ALK-positive OR ALK-rearranged OR ALK-translocated) |  |  |  |
| 9 | (first-line OR front-line OR untreated OR treatment-naive) |  |  |  |
| 10 | (TKI OR tyrosine kinase inhibitors OR ALK-inhibitor OR ALK inhibitor OR crizotinib OR ceritinib OR alectinib OR lorlatinib OR brigatinib OR ensartinib) |  |  |  |
| 11 | 7 AND 8 AND 9 AND 10 | 486 | 85 | 254 |
| 12 | 6 AND 11 |  |  |  |
| 13 | Animals NOT Humans |  |  |  |
| 14 | (news OR comment OR letter OR editorial OR case report OR case reports) |  |  |  |
| 15 | 12 NOT (17 OR 18) | 423 | 81 | 243 |
| 16 | Total number of results (PubMED + EMBASE + Cochrane) | 747 | | |
| 17 | Duplicates identified and deleted | 246 | | |
| 18 | Non duplicated results | 501 | | |

*Supplemental data 1- Table - Search terms and results for each electronic database.*

| **Database/Information source** | **Interface/Address** | **Search Date** |
| --- | --- | --- |
| **Electronic Databases** | | |
| Cochrane Central Register of Controlled Trials (CENTRAL) and Cochrane Database of Systematic Reviews (CDSR) | Cochrane Library/Wiley: https:/[/www.cochra](http://www.cochranelibrary.com/)n[elibrary.com](http://www.cochranelibrary.com/) | 16th Dec 2021 |
| PubMed | https://www.ncbi.nlm.nih.gov/pubmed | 16th Dec 2021 |
| Embase (including MEDLINE) | https://www.embase.com | 17th Dec 2021 |
| **Conference Proceedings / Abstracts from International Meetings** | | |
| American Society of Clinical Oncology (ASCO) | Searched via the Embase database (if the conference was indexed in Embase) or via  the conference website or journal webpages (if free to access) | 14th Dec 2021 |
| European Lung Cancer Conference (ELCC) | Searched via the Embase database (if the conference was indexed in Embase) or via the conference website or journal webpages  (if free to access) | 14th Dec 2021 |
| European Society for Medical Oncology (ESMO) | Searched via the Embase database (if the conference was indexed in Embase) or via  the conference website or journal webpages (if free to access) | 14th Dec 2021 |
| World Conference on Lung Cancer (WCLC) of the International Association for the Study of Lung Cancer (IASLC) | Searched via the Embase database (if the conference was indexed in Embase) or via  the conference website or journal webpages (if free to access) | 15th Dec 2021 |

*Supplemental data 2 - Table - Data sources searched*

| **First author and year of publication** | **N. of OS events: control vs experimental arm** | | **HR (95% CI) and p value for OS analysis (experimental vs control)** | **N./% of ORR events: control vs experimental arm** | | **N./% of DCR events: control vs experimental arm** | | **N./% of any CNSRR events: control vs experimental arm** | | **N./% of measurable CNSRR events: control vs experimental arm** | |
| --- | --- | --- | --- | --- | --- | --- | --- | --- | --- | --- | --- |
| Peters et al 2017 (…) | NA | NA | 0.76 (95% CI: 0.48-1.2). p=0.24 | 114 | 126 | 135 | 138 | 15 | 38 | 11 | 17 |
| Hida et al 2017 (…) | NA | NA | NA | 79% (IRF) 70% (IA) | 92% (IRF) 85% (IA) | 92% (IRF) 88% (IA) | 96% (IRF) 99% (IA) | NA | NA | NA | NA |
| Camidge et al 2020 (…) | 37 | 33 | 0.92 (95% CI: 0.57-1.47). p=0.771 | 62% | 74% | NA | NA | 16% | 66% | 26% | 78% |
| Soria et al 2017 (…) | 59 | 48 | 0.73 (95% CI: 0.5-1.08). p=0.056 | 50 | 137 | NA | NA | 11 | 25 | 6 | 16 |
| Zhou et al 2019 (…) | 13 | 8 | 0.28 (95% CI :0.12-0.68). p=0.0027 | 48 | 114 | 56 | 121 | 5 | 32 | 2 | 16 |
| Horn et al 2021 (…) | 13 | 8 | 0.28 (95% CI: 0.12-0.68). p=0.0027 | 98 | 106 | NA | NA | NA | NA | 4 | 7 |
| Shaw et al 2020 (…) | 28 | 23 | 0.72 (95% CI: 0.41-1.25). p= NA | 85 | 113 | 129 | 135 | 38 | 25 | 3 | 14 |

*Supplemental data 3 - Table - Other outcomes of selected randomized clinical trials in patients with ALK-translocated lung cancer. CI: confidence interval; CNSRR: central nervous system response rate; DCR: disease control rate; HR: hazard ratio; IA: investigator assessed; IRF: independent review facility; NA: not available; ORR: overall response rate; OS: overall survival*

| **First author and year of publication** | **Age subgroups** | **HR (95% CI) for PFS analysis according to age (experimental vs control)** | **Sex subgroups** | **HR (95% CI) for PFS analysis according to sex (experimental vs control)** | **Race subgroups** | **HR (95% CI) for PFS analysis according to race (experimental vs control)** | **Smoking status subgroups** | **HR (95% CI) for PFS analysis according to smoking status (experimental vs control)** | **PS subgroups** | **HR (95% CI) for PFS analysis according to PS (experimental vs control)** | **Baseline CNS involvement subgroups** | **HR (95% CI) for PFS analysis according to baseline CNS involvement at baseline (experimental vs control)** |
| --- | --- | --- | --- | --- | --- | --- | --- | --- | --- | --- | --- | --- |
| Peters et al 2017 (…) | <65 years | 0.48 (0.34-0.7) | Male | 0.61 (0.38-0.98) | Asian | 0.46 (0.28-0.75) | Active smokers | 1.16 (0.35 -3.9) | ECOG 0 | 0.4 (0.21-0.77) | Present | 0.4 (0.25-0.64) |
|  | ≥65 years | 0.45 (0.24-0.87) | Female | 0.39 (0.25-0.6) | Non-Asian | 0.49 (0.32-0.75) | Never smokers | 0.44 (0.29-0.66) | ECOG 1 | 0.48 (0.32-0.71) | Absent | 0.51 (0.33-0.8) |
| Hida et al 2017 (…) | <75 years | 0.34 (0.21-0.56) | Male | 0.35 (0.16-0.77) | All Asian | NA | Active and former smokers | 0.18 (0.08-0.42) | ECOG 0 | NA | Present | 0.08 (0.01-0.61) |
|  | ≥75 years | 0.28 (0.06-1.19) | Female | 0.31 (0.17-0.57) |  |  | Never smokers | 0.5 (0.28-0.89) | ECOG 1 | NA | Absent | 0,39 (0.23-0.64) |
| Camidge et al 2020 (…) | <65 years | 0.43 (0.29-0.65) | Male | 0.46 (0.29-0.74) | Asian | 0.38 (0.22-0.65) | Active smokers | NA | ECOG 0 | 0.25 (0.12-0.52) | Present | 0.25 (0.14-0.46) |
|  | ≥65 years | 0.6 (0.3-1.09) | Female | 0.49 (0.31-0.79) | Non-Asian | 0.54 (0.36-0.83) | Never smokers | 0.46 (0.29-0.73) | ECOG 1 | 0.53 (0.36-0.8) | Absent | 0,65 (0.44-0.97) |
| Soria et al 2017 (…) | <65 years | 0.58 (0.42-0.8) | Male | 0.41 (0.27-0.63) | Asian | 0.66 (0.41-1.06) | Active smokers | 0.48 (0.3-0.77) | WHO 0 | 0.59 (0.37-0.96) | Present | 0.7 (0.44-1.12) |
|  | ≥65 years | 0.45 (0.24-0.86) | Female | 0.63 (0.43-0.93) | Caucasic | 0.44 (0.3-0.66) | Never smokers | 0.56 (0.38-0.8) | WHO 1+2 | 0.52 (0.37-0.74) | Absent | 0,48 (0.34-0.69) |
| Zhou et al 2019 (…) | <65 years | 0.24 (0.14-0.42) | Male | 0.17 (0.09-0.34) | All Asian | NA | Active smokers | 0.01 (0-NE) | ECOG 0 | 0.4 (0.1-1.62) | Present | 0.11 (0.05-0.28) |
|  | ≥65 years | 0,21 (0.04-1.08) | Female | 0.35 (0.16-0.78) |  |  | Never smokers | 0.3 (0.16-0.55) | ECOG 1 | 0.2 (0.11-0.36) | Absent | 0,34 (0.18-0.65) |
| Horn et al 2021 (…) | <65 years (mITT) | 0.42 (0.27-0.64) | Male (mITT) | 0.44 (0.25-0.77) | Asian (mITT) | 0.32 (0.19-0.55) | Active smokers | NA | ECOG 0 | NA | Present | 0.55 (0.3-1.01) |
|  | ≥65 years (mITT) | NA | Female (mITT) | 0.45 (0.25-0.81) | Non-Asian (mITT) | 0.61 (0.34-1.11) | Never smokers | 0.39 (0.23-0.65) | ECOG 1 | NA | Absent | 0,4 (0.23-0.7) |
| Shaw et al 2020 (…) | <65 years | 0.22 (0.13-0.37) | Male | 0.31 (0.18-0.54) | Asian | 0.47 (0.27-0.88) | Active and former smokers | 0.36 (0.2-0.63) | ECOG 0 | NA | Present | 0.2 (0.1-0.43) |
|  | ≥65 years | 0.35 (0.2-0.64) | Female | 0.26 (0.16-0,44) | Non-Asian | 0.19 (0.11-0.32) | Never smokers | 0.24 (0.14-0.4) | ECOG 1 | NA | Absent | 0,32 (0.2-0.49) |

*Supplemental data 4 - Table - Subgroup analysis for PFS in the selected randomized clinical trials. CI: confidence interval; CNS: central nervous system; ECOG: eastern cooperative oncology group; HR: hazard ratio; mITT: modified intention to treat population; NA: not available; NE: not estimable; PFS: progression free survival; PS: performance status; WHO: world health organization*

| **Subgroup analysis on PFS** | | **RR (95% CI)** | **p-value** | **Heterogeneity** |
| --- | --- | --- | --- | --- |
| Age | < 65 | 0.39 (0.29-0.52) | Interaction: p=0.60 | 66.7%; p=0.014 |
|  | ≥ 65 | 0.44 (0.32-0.59) |  | 0%; p=0.69 |
| Gender | Female | 0.41 (0.32-0.53) | Interaction: p=0.75 | 39.7%; p=0.15 |
|  | Male | 0.39 (0.29-0.51) |  | 44.1%; p=0.094 |
| Race | Asian | 0.45 (0.35-0.58) | Interaction: p=0.80 | 14.9%; p=0.35 |
|  | Non-Asian | 0.43 (0.29-0.62) |  | 69.3%; p=0.019 |
| ECOG PS | 0 | 0.42 (0.27-0.64) | Interaction: p=0.97 | 32.7%; p=0.28 |
|  | 1-2 | 0.42 (0.28-0.65) |  | 74.3%; p=0.029 |
| Baseline CNS involvement | Present | 0.30 (0.18-0.51) | Interaction: p=0.17 | 76.4%; p=0.001 |
|  | Absent | 0.45 (0.37-0.55) |  | 22.1%; p=0.30 |
| Smoking status | Active smokers (+ former smokers) | 0.39 (0.28-0.55) | Interaction: p=0.79 | 50.3%; p=0.056 |
|  | Never smokers | 0.41 (0.33-0.51) |  | 29.1%; p=0.21 |

*Supplemental data 5 - Table - Pooled results for the PFS subgroup analysis. CNS: central nervous system; ECOG PS: Eastern Cooperative Oncology Group performance status; RR: relative risk.*


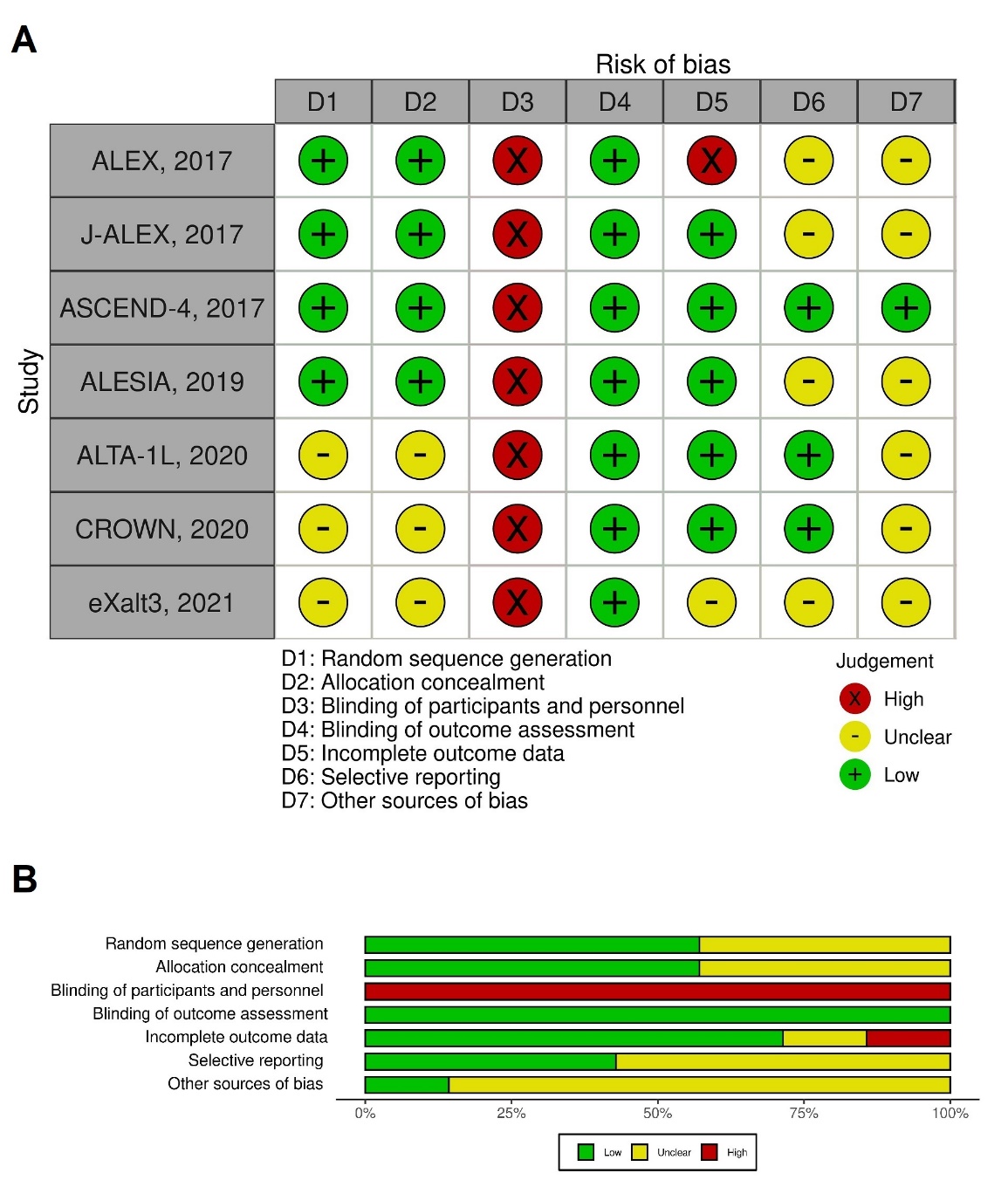


*Supplemental data 6 - Table - Risk of bias graph: review authors' judgements about each risk of bias item presented as percentages across all included studies. A: risk of bias for each study; B: percentages of each risk of bias item.*
